# High Glucose Concentration Impairs 5-PAHSA Activity by Inhibiting AMP-Activated Protein Kinase Activation and Promoting Nuclear Factor-Kappa-B-Mediated Inflammation

**DOI:** 10.3389/fphar.2018.01491

**Published:** 2019-01-07

**Authors:** Yan-Mei Wang, Hong-Xia Liu, Ning-Yuan Fang

**Affiliations:** Department of Geriatrics, Renji Hospital, School of Medicine, Shanghai Jiao Tong University, Shanghai, China

**Keywords:** 5-PAHSA, insulin resistance, inflammation, fatty liver, high glucose

## Abstract

Recently, the endogenous fatty acid palmitic acid-5-hydroxystearic acid (5-PAHSA) was found to increase insulin sensitivity and have anti-inflammatory effects in mice with high-fat diet (HFD)-induced diabetes. However, it is unknown if 5-PAHSA affects glucose and lipid metabolism in db/db mice, which are characterized by extreme hyperglycemia. Here, we aim to determine the effect of continued 5-PAHSA administration on glucose and lipid metabolism in db/db mice. We also used 3T3-L1 cells and HepG2 cells to investigate the mechanism behind this effect. HepG2 cells and 3T3-L1 cells were induced to become models of insulin resistance. The models were used to test the effect of 5-PAHSA on insulin signaling. 5-PAHSA was administered orally to db/db mice for 1 month to assess its effects on glucose and lipid metabolism. We also exposed HepG2 cells to high glucose concentrations to investigate the influence on 5-PAHSA’s effects on hepatic lipid metabolism and inflammation. 5-PAHSA improved glucose uptake and insulin signaling in HepG2 cells and 3T3-L1 cells. However, after 1 month of treatment, 5-PAHSA did not reduce blood glucose levels, but increased inflammation and promoted fatty liver in db/db mice. In HepG2 cells under normal glucose conditions, 5-PAHSA treatment reduced lipogenesis and increased lipid oxidation. Notably, a high glucose concentration in cell media abolished the positive effects of 5-PAHSA treatment. These changes were associated with: decreased phosphorylation of AMP-activated protein kinase (AMPK) and acetyl-CoA carboxylase (ACC); upregulation of sterol-regulatory element-binding protein 1c (SREBP1c), and fatty acid synthase (FAS); and downregulation of carnitine palmitoyltransferase 1 (CPT1). Besides, the anti-inflammatory effect of 5-PAHSA was also impaired by high glucose conditions. Thus, high glucose concentrations impaired 5-PAHSA action by inhibiting the AMPK signaling pathway and promoting nuclear factor-kappa-B (NF-κB) mediated inflammation.

## Introduction

Type 2 diabetes mellitus (T2DM) is a disease that affects more than 400 million people worldwide (Nathan, 2015). The medical and economic burdens of diabetes present an important public health challenge for the developed and the developing world (American Diabetes Association, 2013; Le et al., 2013; Seuring et al., 2015; Bao et al., 2017). Further, there is an urgent need for safe and effective interventions for T2DM because most currently used medications for long-term T2DM treatment have side effects, such as hypoglycemia, weight gain, and gastrointestinal reactions (Stein et al., 2013).

There is a strong association between T2DM and dyslipidemia, as >70% of patients with T2DM develop non-alcoholic fatty liver disease (NAFLD) with the inflammatory complication, non-alcoholic steatohepatitis (NASH) (Williams et al., 2011; Loomba et al., 2012). Several studies have demonstrated that elevated levels of free fatty acids play a causative role in metabolic syndrome (Devaraj et al., 2008; Sun and Chen, 2010; Perry et al., 2015). However, Yore et al. (2014) reported recently that the endogenous fatty acid, 5-PAHSA, had favorable metabolic effects via binding and activating its G-protein-coupled receptor 120 (GPR120). Consistent downregulation of 5-PAHSA was found in all adipose depots and the serum of insulin-resistant mice, as well as in the white adipose tissue and serum of insulin-resistant humans. Acute oral administration of 5-PAHSA in insulin-resistant high-fat diet (HFD)-fed mice lowered basal glycemia and improved glucose tolerance (Yore et al., 2014). Moreover, chronic 5-PAHSA treatment improved insulin sensitivity in chow- and HFD-fed mice (Syed et al., 2018). Besides, 5-PAHSA might also reduce inflammatory cytokine production from immune cells and ameliorate adipose inflammation in obesity.

Discovery of the anti-diabetic and anti-inflammatory effects of 5-PAHSA make it a promising candidate for further research and drug development. However, when considering clinical applications, the effect of repeated and continuous administration of 5-PAHSA for diabetic metabolic disorders should be better understood.

Leptin receptor deficient db/db mice are widely used as a diabetic model for T2DM research (Wang et al., 2014). The db/db mouse is the most popular animal model used by pharmaceutical companies to test blood glucose lowering agents, insulin sensitizers, insulin secretagogues, and anti-obesity agents (Reed and Scribner, 1999). Since the effect of 5-PAHSA treatment on db/db mice has not been characterized, one aim of this study was to investigate the effects of repeated 5-PAHSA treatment on glucose and lipid metabolism in db/db mice. We found that 1 month of 5-PAHSA administration had no beneficial effect on glucose metabolism in db/db mice. Moreover, we found that the course of 5-PAHSA treatment promoted hepatic fatty infiltration and inflammatory responses in these mice. The mechanisms governing these negative effects of 5-PAHSA treatment, particularly in the liver, are not fully understood. Given that high blood glucose levels are characteristic of db/db mice, we hypothesized that hyperglycemia may alter the effects of 5-PAHSA treatment and tested this hypothesis in human liver-derived HepG2 cells. Thus, the other aim of this study was to study the role of hyperglycemia in the impairment of 5-PAHSA action.

## Materials and Methods

### Reagents

Radio-immunoprecipitation assay (RIPA) buffer, Dulbecco’s Modified Eagle’s Medium (DMEM), TRIzol reagent, fetal bovine serum (FBS), penicillin/streptomycin, phenylmethylsulfonyl fluoride (PMSF), and Halt protease and phosphatase inhibitor cocktail were obtained from Thermo Fisher Scientific (Waltham, MA, United States). D-glucose, bovine serum albumin (BSA), insulin and Dimethylsulfoxide (DMSO) were obtained from Sigma-Aldrich (St. Louis, MO, United States). Phosphate-buffered saline (PBS) was obtained from GE Healthcare Life Sciences (Beijing, China). The bicinchoninic acid (BCA) protein assay and primary antibody dilution buffer were obtained from Beyotime (Nanjing, China). Antibodies against insulin receptor substrate 1 (IRS1), pThr896-IRS1, insulin receptor substrate 2 (IRS2), pSer731-IRS2, protein kinase B (Akt), pSer473-Akt, GPADH, AMP-activated protein kinase alpha (AMPKα), pThr172-AMPKα, acetyl-CoA carboxylase (ACC), pSer79-ACC, carnitine palmitoyltransferase 1 (CPT1), fatty acid synthase (FAS), IκB alpha, pSer36-IκB alpha, NF-κB, pSer536-NF-κB, and β-actin were obtained from Abcam (Cambridge, MA, United States), and the antibody against sterol-regulatory element-binding protein 1c (SREBP1c) was obtained from Santa Cruz (Dallas, TX, United States).

### Synthesis and Verification of 5-PAHSA

Detailed information on synthesis and verification of 5-PAHSA is outlined in the Supplementary Information.

### Cell Culture and Treatments

3T3-L1 cells and human hepatoma HepG2 cell line were obtained from the Chinese Academy of Medical Sciences and Peking Union Medical College (Beijing, China). 3T3-L1 cells were grown in DMEM supplemented with 10% FBS, 200 U/ml penicillin, and 200 U/ml streptomycin in 5% CO_2_ humidified atmosphere at 37°C until confluence. Two days after confluence, to induce adipocyte differentiation, cells were incubated for 48 h in DMEM supplemented with 10% FBS containing 500 μM 3-isobutyl-1-methylxanthine (IBMX), 0.25 μM dexamethasone, and 10 μg/ml insulin. Then the cells were maintained in culture medium supplemented with insulin only, which were changed every 2 days until complete differentiation. To induce insulin resistance, mature 3T3-L1 cells were treated with recombinant mouse tumor necrosis factor (TNF)-α (4 ng/ml). Media was changed daily for TNF-α treatment, for a total incubation time of 4 days. For 5-PAHSA treatment, 3T3-L1 cells were treatment with 5-PAHSA (20 μM) for 2 days, and then harvested for RNA or protein isolation.

We cultured HepG2 cells in DMEM, with either a normal glucose concentration (5.5 mM D-glucose) or a high glucose concentration (30 mM). To induce insulin resistance, HepG2 cells were treatment with high insulin (100 nM). Media was changed daily for a total incubation time of 3 days. For 5-PAHSA treatment, HepG2 cells were treated with 5-PAHSA (20 μM) for 2 days, and then harvested for RNA or protein isolation.

### Immunofluorescence Staining

Immunofluorescence staining was conducted based on standard procedures for the Glut4 membrane translocation analysis. Briefly, cells were blocked with 5% BSA for 30 min at room temperature with membrane rupture treatment by Triton to detect total Glut4 or without membrane rupture to determine membrane distribution. Cells were incubated at 4°C with anti-Glut4 antibody overnight. Equal PBS was added instead of Glut4 as a negative control. The Cy3-conjugated secondary antibody was applied to the samples at room temperature for 1 h. After washing with PBS, images were immediately captured under an immunofluorescence microscope.

### Glucose Uptake Assay

Cells in 96 well dishes were washed twice with PBS and incubated with 100 μl KRPH/2% BSA for 40 min. Prepare sample background controls, insulin stimulated cells and non-stimulated control samples. (1) Sample background control (untreated) cells: Do not add insulin and 2-deoxyglucose (2-DG). (2) Insulin stimulated cells: KRPH/2% BSA contained with 10 μM insulin for 20 min and add 10 μl of 10 mM 2-DG for 20 min. (3) Non-stimulated control samples: Non-insulin stimulated cells, but add 10 μl of 10mM 2-DG for 20 min. Prepare Reaction Mix A and add in all samples. And incubate for 1 h. Add 90 μl Extraction buffer in each well and heat at 90°C for 40 min. Prepare Reaction Mix B fresh and add 38 μl in all wells. Measure output OD at 412 nm wavelength on a microplate reader in a kinetic mode, every 2–3 min, at 37°C protected from light.

### Animals

All animal experiments were approved by Fudan University Animal Care and Use Committee. Male db/db and wild-type (WT) C57BL6/J mice were purchased from the Model Animal Research Center of Nanjing University (Nanjing, China). At 3 weeks of age, the C57BL/6 mice were given high fat diet (HFD, Shanghai SLAC Company) for 20 weeks. Mice with random blood glucose >11.1 mmol/L were considered as insulin-resistant mice. Individual db/db mouse weighed 40 ± 5 g, WT mouse weighed 28 ± 2 g and HFD-induced insulin-resistant mouse weighed 32 ± 2 g. All mice were housed in colony cages with *ad libitum* access to food and water. Mice were kept on a 12/12 h light/dark cycle in a temperature-controlled environment.

### Groups and Intervention

Mice were divided into six groups: db/db control group (db/db/C); db/db+5-PAHSA (50 mg/kg per day) group (db/db/L); db/db+5-PAHSA (150 mg/kg per day) group (db/db/H); WT control group (WT/C); WT+5-PAHSA (50 mg/kg per day) group (WT/L). WT+5-PAHSA (150 mg/kg per day) group (WT/H) (*n* = 5 for each group). For glucose metabolism studies, we added HFD-induced diabetic mice (control; HFD plus 50 mg/kg 5-PAHSA, *n* = 5 mice) to the above two groups.

We administered 5-PAHSA by gavage once a day for 1 month. The control mice were given with the same volume of vehicle [50% polyethylene glycol (PEG) 400, 0.5% Tween-80, 49.5% H_2_O] at the corresponding time points.

### Oral Glucose Tolerance Tests (OGTT)

We performed OGTT 5 h after food removal in awake mice. At 4.5 h after food removal (0.5 h before initiation of the OGTT), we gavaged mice with 50 mg/kg/150 mg/kg 5-PAHSA, or with an equivalent volume of vehicle. We gave mice 1 g/kg glucose by gavage 30 min after administration of 5-PAHSA or vehicle and monitored blood glucose over a 2 h period. Before the 5-PAHSA gavage and 5 min after the glucose gavage, we bled mice from the tail vein using heparin coated capillary tubes and determined blood glucose by Accu-Check active bands (Roche Diagnostics) (*n* = 5 mice for each group).

### Measurement of Blood Glucose

We collected blood from the tail vein of each mouse using heparin coated capillary tubes. We measured blood glucose with Accu-Check active bands (Roche Diagnostics) (*n* = 5 mice for each group).

### Staining of Mice Liver Specimens

We fixed liver specimens in 10% formalin, embedded in paraffin, sectioned the tissue (4 μm), and then stained sections with hematoxylin-eosin. We blindly assessed infiltration of inflammatory cells in liver on four random fragments from different areas of each liver (*n* = 5 mice for each group).

### Enzyme-Linked Immunosorbent Assay (ELISA)

We collected blood from the abdominal aorta of mice at the time of sacrifice. Serum C-reactive protein (CRP), TNF-α, interleukin (IL)-1α, and insulin levels were measured by ELISA kit (Sigma-Aldrich, St. Louis, MO, United States), according to the manufacturer’s instructions (*n* = 5 mice for each group).

We extracted hepatic proteins from 50 mg of mouse liver homogenate by homogenization in 1.5 mL of phosphate-buffered saline (PBS) using TissueLyser (Qiagen, CA, United States). We centrifuged the homogenate for 10 min at 1000 g and collected the protein from the lower phase. We quantified CRP and TNF-α in the liver with ELISA (Sigma-Aldrich, St. Louis, MO, United States) (*n* = 5 mice for each group).

Levels of interleukin 6 (IL-6) and monocyte chemotactic protein 1 (MCP1) in HepG2 cells-conditioned medium were measured using ELISA (Sigma-Aldrich, St. Louis, MO, United States).

### Gene Expression Analysis

TRIzol reagent was used to extract total RNA from HepG2 cells. A total of 1 μg RNA was subjected to reverse transcription using the PrimeScript^TM^ RT Reagent kit (Takara, Shiga, Japan). Gene expression was evaluated by Quantitative reverse transcription PCR (qPCR) analysis using SYBR Green reagents (SYBR^®^ Premix Ex Taq^TM^) and the LightCycler^®^ 480 Real-Time PCR System (Roche Diagnostics, Basel, Switzerland). The primers for qPCR of HepG2 cells are shown in Table 1.

**Table 1:** The primers for qPCR of HepG2 cells.

Primer	Direction	Sequence (5′–3′)
β-actin	Forward	AGC CTT GTA GGT ACC CAA CC
	Reverse	TCC CAC TCA CCT GAG GTG CTG AA
FAS	Forward	AGG TGG TGA TAG CCGGTA TGT
	Reverse	TGG GTA ATC CAT AGA GCC CAG
CPT1	Forward	CGA TCA TCA TGA CTA TGC GCT ACT
	Reverse	GCC GTG CTC TGC AAA CAT C
SREBPlc	Forward	CAC CGT TTC TTC GTG GAT GG
	Reverse	CCC GCA GCA TCA GAA CAG C
MCP1	Forward	CGC CTC CAG CAT GAA AGT CT
	Reverse	GGA ATG AAG GTG GCT GCT ATG
IL-6	Forward	GGT ACA TCC TCG ACG GCA TCT
	Reverse	GTG CCT CTT TGC TGC TTT CAC

### Oil Red O Staining

Cells were washed with PBS, fixation in 10% formalin for 20 min at room temperature, and further washing with PBS. A mixture of Oil Red O solution and water with ratio of 3:2 was layered onto cells for 60 min, followed by washing three times with deionized water. Images were subsequently captured under a microscope.

### Intracellular Triglyceride and Cholesterol Content Assay

We pre-incubated HepG2 cells in a 6-well cell culture plate for 72 h. The cells were cultured in DMEM containing either normal glucose or high glucose, supplemented with 5-PAHSA or vehicle control. After a 72 h incubation, we collected and centrifuged cells at 1000 rpm for 10 min. Cell pellets were washed once with PBS, resuspended in 400 μL PBS buffer, and transferred to a microsmashing tube for ultrasonication (ultrasonic output power of 400 w, intermission/ultrasonication time of 30 s/5 s, total extraction time of 15 s). After ultrasonication, we determined the concentration of cellular triglyceride using an EnzyChrom^TM^ triglyceride assay kit (Bioassay Systems, Hayward, CA, United States) and normalized the measured value to the protein concentration, according to the manufacturer’s protocol.

### Western Blot Analysis

We applied equal amounts of protein (15 μg/lane) to a 10% SDS-polyacrylamide gel electrophoresis and transferred to a polyvinylidene fluoride (PVDF) membrane (Bio-Rad, CA, United States). We blocked membranes with 5% non-fat dry milk in Tris-buffered saline (TBS) (Amersham Biosciences, Uppsala, Sweden) containing 0.05% Tween-20 (T-TBS) (Bio-Rad, CA, United States) and then incubated overnight at 4°C with antibodies to reveal the expression of them. Then we washed the membranes with T-TBS and incubated with specific secondary antibodies for 1 h. We visualized peroxidase activity with an enhanced chemiluminescence substrate system (ECL; Santa Cruz Biotechnology, Santa Cruz, CA, United States) and quantified the bands using Quantity One (Bio-Rad, CA, United States).

### Statistical Analysis

All data are representative of at least three different experiments and are expressed as the mean ± standard error (SE). All data were analyzed using GraphPad Prism software (GraphPad Software Inc., San Diego, CA, United States). We used the analysis of variance (ANOVA) to compare the differences between multiple groups. The non-paired *t*-test was used to analyze two groups after the homogeneity of variance test. We considered differences to be statistically significant at *p* < 0.05.

## Results

### Effects of 5-PAHSA on Insulin Resistance *in vitro*

**FIGURE 1:**
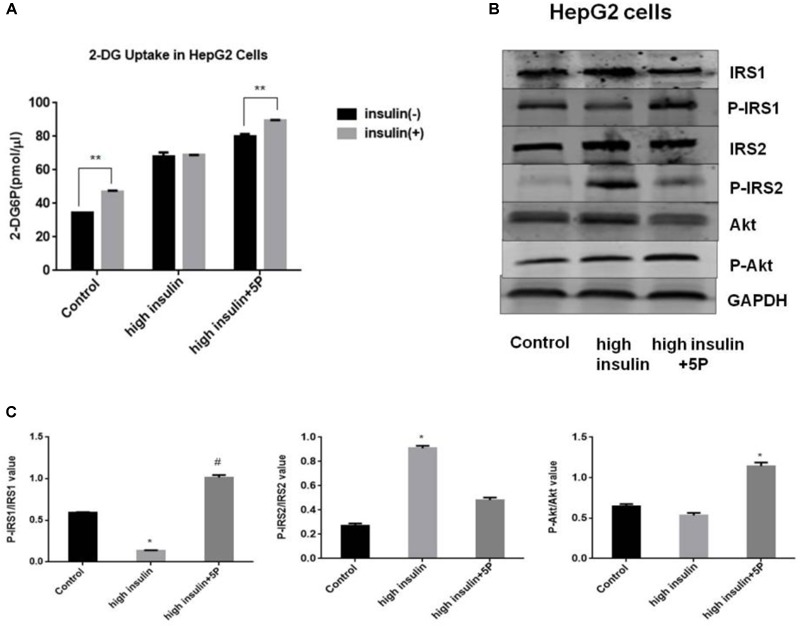
Effects of 5-PAHSA treatment on glucose uptake and insulin signaling pathway in HepG2 cells. **(A)** Rates of glucose transport in HepG2 cells. Basal glucose transport (black) and insulin stimulated glucose transport (gray) are shown. Cells were untreated, treated with high insulin or high insulin plus 5-PAHSA. Basal rate refers to the rate of glucose transport in the absence of insulin. Insulin-stimulated rate was calculated as the rate of transport in the presence of insulin minus the basal rate. ^∗∗^*p* < 0.01 versus basal rate (*t*-test). **(B)** Insulin signaling was examined by western blot analysis in HepG2 cells. Cells were untreated, treated with high insulin or high insulin plus 5-PAHSA. **(C)** Quantification of western blotting. ^∗^*p* < 0.05 versus control, ^#^*p* < 0.05 versus high insulin (one-way ANOVA). Data are representative of at least three different experiments. All data represent means ± standard error (SE).

To test the effects of 5-PAHSA on insulin resistance, HepG2 cells and 3T3-L1 cells were treated with high insulin and TNF-α, respectively, to become experimental models of insulin resistance (IR). The models were assessed by the ability of insulin to stimulate glucose uptakes. In HepG2 cells, high insulin treatment impaired the action of insulin to stimulate glucose uptake, indicating the successful establishment of IR model using HepG2 cells. However, the defect could be rescued by 5-PAHSA treatment (Figure 1A). We also examined the effect of 5-PAHSA on various parameters of insulin signaling. The results showed that treatment with high insulin decreased levels of insulin-stimulated IRS1 phosphorylation at Thr896 and Akt phosphorylation at Ser473, increased IRS2 phosphorylation at Ser731, whereas co-treatment with 5-PAHSA largely prevented this actions (Figures 1B,C).

**FIGURE 2:**
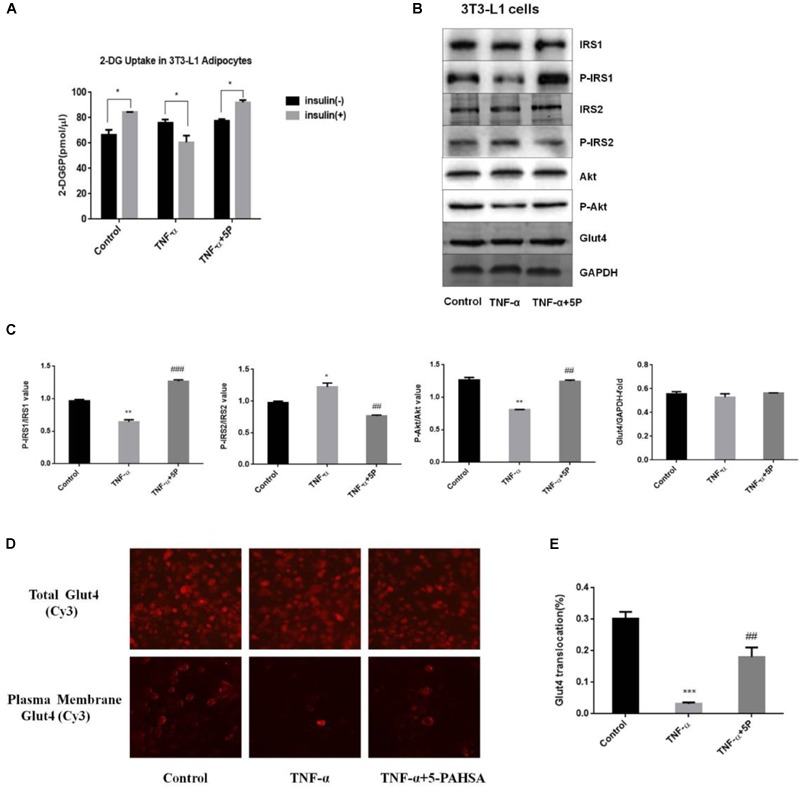
Effects of 5-PAHSA treatment on glucose uptake and insulin signaling pathway in 3T3-L1 cells. **(A)** Rates of glucose transport in 3T3-L1 cells. Basal glucose transport (black) and insulin stimulated glucose transport (gray) are shown. Cells were untreated, treated with TNF-α alone or TNF-α plus 5-PAHSA. ^∗^*p* < 0.05 versus basal rate (*t*-test). **(B)** Insulin signaling was examined by western blotting analysis in 3T3-L1 cells. Cells were untreated, treated with TNF-α alone or TNF-α plus 5-PAHSA. **(C)** Quantification of western blotting. ^∗^*p* < 0.05, ^∗∗^*p* < 0.01 versus control, ^##^*p* < 0.01, ^###^*p* < 0.001 versus TNF-α (one-way ANOVA). **(D)** Glut4 plasma membrane translocation in 3T3-L1 adipocytes treated with TNF-α alone or together with 5-PAHSA. **(E)** Quantification of Glut4 translocation in **(D)**. ^∗∗∗^*p* < 0.001 versus control, ^##^*p* < 0.01 versus TNF-α (one-way ANOVA). Data are representative of at least three different experiments. All data represent means ± standard error (SE).

In 3T3-L1 adipocytes, insulin-dependent glucose uptake was decreased by TNF-α treatment. The results showed the successful establishment of IR model using 3T3-L1 adipocytes. However, the defect in insulin action was reversible by 5-PAHSA treatment (Figure 2A). TNF-α treatment decreased levels of insulin-stimulated phosphorylation on IRS1 (Thr896) and Akt (Ser473), increased phosphorylation on IRS2 (Ser731), but 5-PAHSA treatment largely prevented this effects (Figures 2B,C). TNF-α alone or together with 5-PAHSA had no effect on total Glut4 levels (Figures 2B–D). However, the expression levels of Glut4 on cell surface were decreased in TNF-α treated 3T3-L1 adipocytes compared with control based on immunofluorescence detection. In contrast, 5-PAHSA treatment significantly increased Glut4 plasma membrane translocation (Figure 2E). Together, our results indicated that 5-PAHSA can significantly reduce high insulin- and TNF-α-induced insulin resistance in HepG2 cells and 3T3-L1 cells.

### 5-PAHSA Treatment Improved Glucose Tolerance in HFD-Induced Insulin-Resistant Mice, While Had No Effect on Glucose Tolerance, Blood Glucose, Insulin Levels or Body Weight in db/db Mice

**FIGURE 3:**
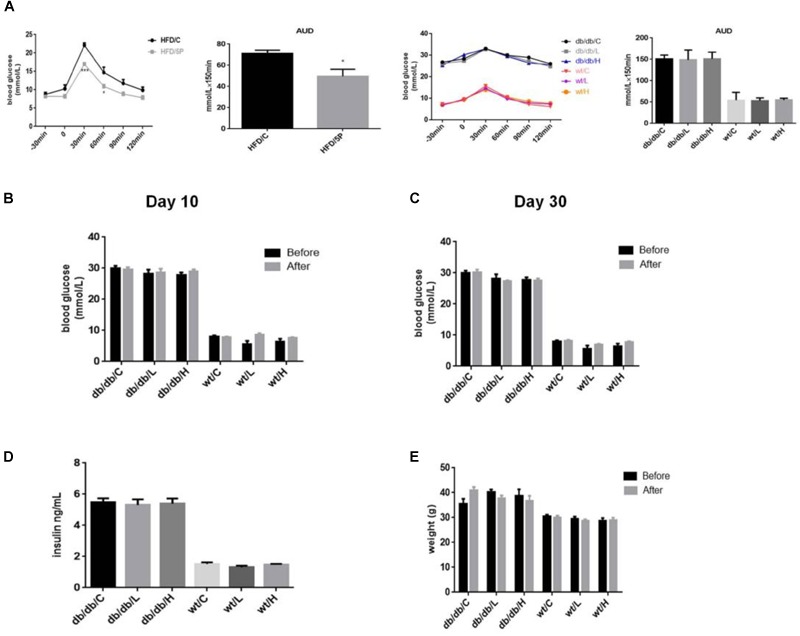
5-PAHSA treatment improved glucose tolerance in HFD-induced insulin-resistant mice, while had no effect on glucose tolerance, blood glucose, insulin levels or body weight in db/db mice. All mice were divided randomly as follows: control db/db group (db/db/C), db/db plus low-dose 9-PAHSA group (50 mg/kg per day; db/db/L), db/db plus high-dose 9-PAHSA group (150 mg/kg per day; db/db/H), control WT group (WT/C), WT plus low-dose 9-PAHSA group (50 mg/kg per day; WT/L), and WT plus high-dose 9-PAHSA group (150 mg/kg per day; WT/H); HFD control and HFD plus 50 mg/kg 5-PAHSA. **(A)** HFD-induced insulin-resistant mice, db/db mice and WT mice were gavaged with 5-PAHSA or a vehicle control 4.5 h after food removal. After 30 min, an oral glucose tolerance test (OGTT) was performed. Area under the curve (AUC) was calculated from –30 to 120 min. ^∗^*p* < 0.05 (t test), ^∗∗∗^*p* < 0.001 (two-way ANOVA) versus HDF/C. **(B,C)** Changes in blood glucose of db/db mice and WT mice after 10 and 30 days of 5-PAHSA treatment. **(D)** Serum insulin levels in db/db mice and WT mice after 30 days of 5-PAHSA treatment. **(E)** Body weight changes in db/db mice and WT mice after 30 days of 5-PAHSA treatment. *n* = 5 mice per group. All data represent means ± standard error (SE).

We nest sought to extend these observations from cellular models to *in vivo* models of insulin resistance, the HFD-induced insulin resistant mice and the leptin receptor-deficient db/db mice. To test whether administration of 5-PAHSA can improve glucose tolerance, an OGTT was performed in insulin-resistant HFD-fed mice and db/db mice. The results showed that 5-PAHSA treatment improved glucose tolerance with a reduced area under the glucose excursion curve in 5-PAHSA treated HFD-fed mice. However, low dose (50 mg/kg) and high dose (150 mg/kg) of 5-PAHSA treatment did not improve glucose tolerance in db/db mice (Figure 3A). Then we further test whether continuing 5-PAHSA treatment would affect glucose metabolism in db/db mice, blood glucose levels were tested after 5-PAHSA treatment for 10 and 30 days in db/db mice. There was still no significant differences in blood glucose levels as compared to before treatment (Figures 3B,C). To determine whether continuing administration of 5-PAHSA influenced insulin secretion, serum insulin levels were tested after 30 days of low dose and high dose of 5-PAHSA treatment. They had no effect on insulin secretion (Figure 3D). Further, 30 days of 5-PAHSA treatment did not significantly change body weight (Figure 3E) or food intake (data not shown) in db/db mice. Besides, 5-PAHSA treatment also had no effects on glucose metabolism in WT mice.

### Chronic 5-PAHSA Administration Induced Liver Steatosis and Secretion of Inflammatory Factors in db/db Mice

**FIGURE 4:**
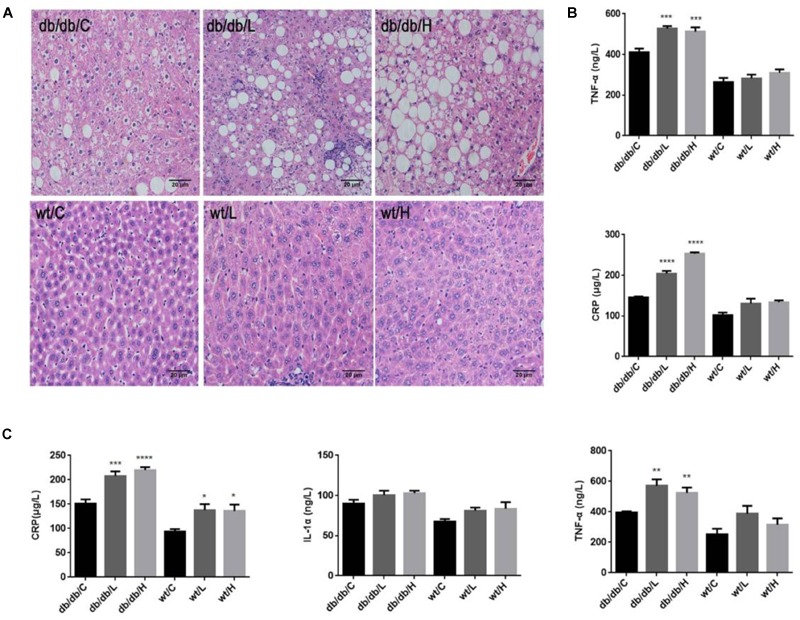
Effects of repeated 5-PAHSA administration on hepatic lipid accumulation and inflammatory cytokines secretion in db/db mice and WT mice. **(A)** Hematoxylin and eosin staining of hepatic tissue sections after 30 days of 5-PAHSA treatment. Pictures were taken at 200× magnification and show: db/db/C, Mild steatosis; db/db/L, Severe steatosis and inflammatory cells infiltration; db/db/H, Severe steatosis; WT/C, None steatosis; WT/L, None steatosis; WT/H, None steatosis. **(B)** TNF-α and CRP levels in liver after 30 days of 5-PAHSA treatment. **(C)** Levels of serum inflammatory cytokines, including CRP, IL-1α and TNF-α after 30 days of 5-PAHSA treatment. ^∗^*p* < 0.05, ^∗∗^*p* < 0.01, ^∗∗∗^*p* < 0.001, ^∗∗∗∗^*p* < 0.0001 versus db/db/C or WT/C. *n* = 5 mice per group. Data represented means ± standard error (SE).

Since we didn’t observe hypoglycemic action of chronic 5-PAHSA treatment in db/db mice, we further investigated whether 5-PAHSA treatment could affect lipid metabolism in the livers of db/db mice. In low dose and high dose of 5-PAHSA treated db/db mice, more severe liver steatosis was observed, compared with vehicle control. And these effects of 5-PAHSA were not be depend on dosage (Figure 4A). In addition, the infiltration of inflammatory cells was also been seen in livers of low dose of 5-PAHSA-treated db/db mice (Figure 4A). In addition, no significant differences were observed in 5-PAHSA-treated WT mice and vehicle control.

We then tested the secretion of liver inflammatory factors, CRP and TNF-α. The protein levels of CRP and TNF-α were significantly increased in 5-PAHSA-treated db/db mice than vehicle control (Figure 4B). Similarly, serum levels of IL-1α, TNF-α, and CRP were significantly higher in 5-PAHSA-treated db/db mice than vehicle control (Figure 4C). However, the effects of 5-PAHSA were not dose-dependent. Likewise, 5-PAHSA had no effect on inflammatory factors in WT mice (Figures 4B,C). These results suggest that chronic 5-PAHSA administration may induce systemic inflammatory responses in db/db mice.

### High Glucose Impaired the Effects of 5-PAHSA on Lipid Metabolism in HepG2 Cells

**FIGURE 5:**
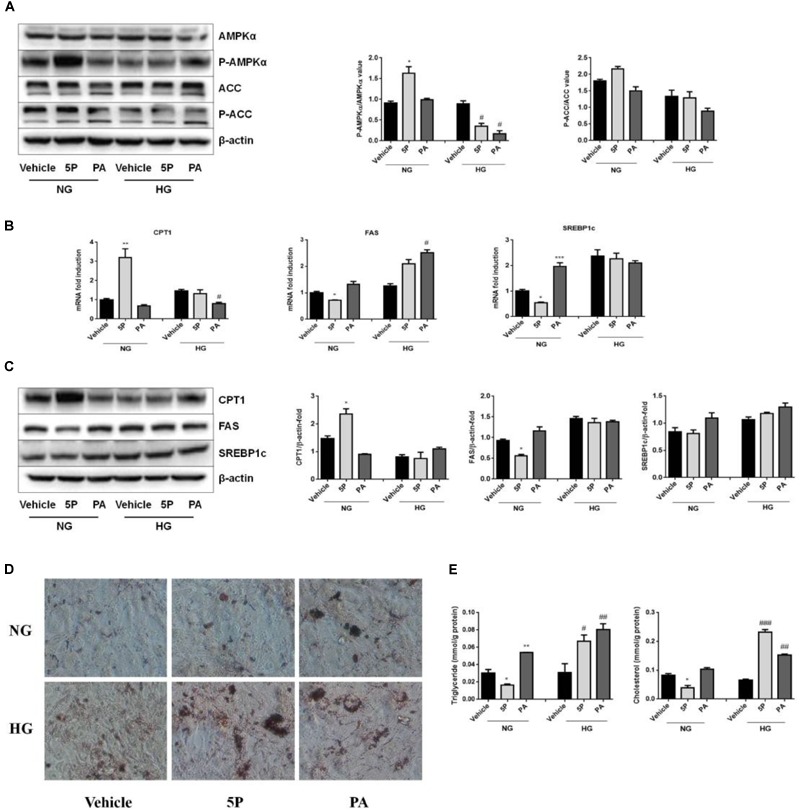
Effects of 5-PAHSA treatment on lipid metabolism under normal and high glucose conditions in HepG2 cells. HepG2 cells were cultured with normal glucose (NG) or high glucose (HG) for 72 h. Cells were untreated (vehicle), treated with 5-PAHSA (5P) or palmitic acid (PA). **(A)** Western blotting analysis of protein expression, including total AMPKα and pThr172-AMPKα, total ACC and pSer79-ACC. **(B)** The mRNA expression of CPT1, FAS, and SREBP1c were evaluated by qPCR. **(C)** Western blotting analysis of protein expression, including CPT1, FAS, and SREBP1c. **(D)** Lipid accumulation was detected by Oil Red O staining (magnification, 200×). **(E)** The levels of intracellular triacylglycerols and cholesterol in HepG2 cells were tested by ELISA. ^∗^*p* < 0.5, ^∗∗^*p* < 0.01, ^∗∗∗^*p* < 0.001 versus Vehicle under normal glucose condition; ^#^*p* < 0.5, ^##^*p* < 0.01, ^###^*p* < 0.001 versus vehicle under high glucose condition (one-way ANOVA). Data are representative of at least three different experiments. Data represent means ± standard error (SE).

In HFD-induced insulin-resistant mice, 5-PAHSA improves glucose metabolism (Yore et al., 2014). In the study, we also found that 5-PAHSA treatment improved glucose tolerance and insulin sensitivity in HFD-fed mice, HepG2 cells and 3T3-L1 cell (Figures 1, 2). However, 5-PAHSA treatment did not metabolically benefit db/db mice. Based on it, we hypothesized that hyperglycemia condition in db/db mice impaired 5-PAHSA effects on metabolism. To test this hypothesis, we exposed HepG2 cells to either a normal level of glucose or a high level of glucose. To determine whether the effects of 5-PAHSA on metabolic parameters are distinct from effects of ordinary free fatty acids, we performed similar studies with PA since 5-PAHSA is made up of palmitate and hydroxystearic acid. We found that 5-PAHSA treatment enhanced phosphorylation levels of key lipid metabolism enzymes in normal glucose conditions compared to control and PA treatment. As expected, 5-PAHSA treatment increased phosphorylation of AMPKα on Thr172 (Figure 5A). Consequently, phosphorylation of ACC on Ser79, an AMPKα target, also increased slightly (Figure 5A), indicating that 5-PAHSA treatment inactivated this rate-limiting enzyme of fatty acid synthesis. In high glucose conditions, 5-PAHSA treatment of HepG2 cells decreased phosphorylation levels of AMPKα, but had no significant influence on ACC phosphorylation (Figure 5A). PA treatment had similar effects (Figure 5A).

In normal glucose conditions, 5-PAHSA treatment upregulated mRNA and protein expression of CPT1, downregulated mRNA and protein expression of FAS or SREBP1c compared to control and PA treatment (Figures 5B,C). These effects of 5-PAHSA were lost when cells were subjected to high glucose conditions (Figures 5B,C).

As 5-PAHSA treatment in the presence of high glucose increased mRNA and protein levels of genes involved in lipid synthesis and reduced fatty acid oxidation, we further investigated whether this treatment promoted lipid accumulation in HepG2 cells. Oil Red O staining showed no signs of lipid accumulation among control group, 5-PAHSA group and PA group incubated with normal glucose. In contrast, in the high glucose conditions, 5-PAHSA and PA treatment showed significantly lipid accumulation compared with control group (Figure 5D). In addition, quantification of lipid content in HepG2 cells revealed that, in the normal glucose conditions, 5-PAHSA decreased intracellular levels of triglycerides and cholesterol in HepG2 cells, as compared to control and PA treatment (Figure 5E). In the high glucose conditions, 5-PAHSA treatment significantly increased intracellular levels of triglycerides and cholesterol, as effectively as PA treatment (Figure 5E).

### High Glucose Impaired the Anti-inflammatory Effects of 5-PAHSA in HepG2 Cells

**FIGURE 6:**
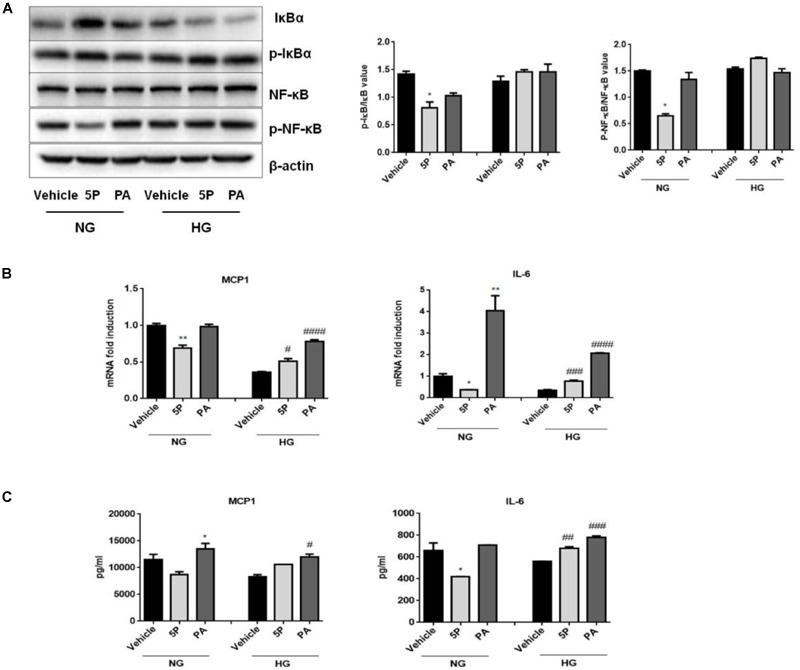
Effects of 5-PAHSA treatment on the NF-κB-mediated signaling pathway under normal and high glucose conditions in HepG2 cells. **(A)** Western blotting analysis of expression of total IκBα and pSer36-IκBα, total NF-κB and pSer536-NF-κB. **(B)** The mRNA expression of MCP1 and IL-6 were evaluated by qPCR. **(C)** The protein levels of MCP1 and IL-6 were tested by ELISA. ^∗^*p* < 0.5, ^∗∗^*p* < 0.01 versus vehicle under normal glucose condition; ^#^*p* < 0.5, ^##^*p* < 0.01, ^###^*p* < 0.001, ^####^*p* < 0.0001 versus vehicle under high glucose condition (one-way ANOVA). Data are representative of at least three different experiments. Data represent means ± standard error (SE).

5-PAHSA treatment promoted the secretion of liver and serum inflammatory factors in db/db mice. To determine whether 5-PAHSA treatment increased inflammation induced by high glucose concentrations, HepG2 cells were incubated with either normal or high glucose concentrations in the absence or presence of 5-PAHSA. In the normal glucose conditions, 5-PAHSA treatment inhibited phosphorylation of IκBα and NF-κB, prevented IκBα degradation and then NF-κB activation, as compared to control and PA treatment (Figure 6A). Interestingly, all of these effects of 5-PAHSA were completely abrogated by high glucose concentrations (Figure 6A). Consistent with this, 5-PAHSA treatment decreased mRNA and protein levels of MCP1 and IL-6 in normal glucose compared with control and PA treatment (Figures 6B,C). Similar to PA, 5-PAHSA increased mRNA and protein levels of MCP1 and IL-6 in high glucose conditions (Figures 6B,C). These results indicated that high glucose impaired the anti-inflammatory function of 5-PAHSA.

## Discussion

In the study, we found that the HFD-induced insulin-resistant mice exhibited improved glucose tolerance after 5-PAHSA treatment, evidenced by reduced area under the glucose excursion curve. Moreover, 5-PAHSA improved insulin sensitivity in 3T3-L1 cells and HepG2 cells. Specifically, 5-PAHSA treatment significantly increased insulin stimulated glucose uptake, thus improved insulin resistance induced by high insulin/TNF-α. Besides, 5-PAHSA treatment increased IRS1 phosphorylation at Thr896 and Akt phosphorylation at Ser473, two signaling proteins correlated with increases in glucose uptake. And 5-PAHSA treatment decreased IRS2 phosphorylation at Ser731, which related with insulin resistance. 5-PAHSA treatment also enhanced Glut4 translocation in 3T3-L1 cells, leading to increased uptake of glucose. Consistent with these findings, previous studies have reported acute oral administration of 5-PAHSA to insulin-resistant HFD-fed mice lowered basal glycemia and improved glucose tolerance (Yore et al., 2014). Chronic 5-PAHSA treatment also improved insulin sensitivity and glucose tolerance in chow- and HFD-fed mice (Syed et al., 2018). However, Pflimlin et al. (2018) challenge these findings that 5-PAHSA improves glucose control *in vivo*. They reported that acute and repeated treatment with 5-PAHSA in insulin-resistant diet-induced obese mice did not improve the metabolic status. Likewise, we found that repeated administration of 5-PAHSA for 1 month didn’t metabolically benefit db/db mice.

The important methodological issues that may contribute to the different results among these researches. The db/db mouse is one of the most widely used animal models in T2DM research (Wang et al., 2014). However, unlike HFD-induced insulin-resistant mice, the diabetic characteristics of db/db mice derive from mutation of leptin receptor genes. In db/db mice at 10 weeks of age, fasting blood glucose levels can reach ∼600 mg/dl in comparison to ∼150 mg/dl in control mice (Han et al., 2008), indicating extreme hyperglycemia. High levels of glucose may impair 5-PAHSA signaling pathway in db/db mice. Our *in vitro* results also confirmed this hypothesis.

Here, we showed that 5-PAHSA treatment reduced lipogenesis and increased fatty acid oxidation in HepG2 cells in normal glucose conditions. In contrast, 5-PAHSA treatment under high glucose conditions promoted fatty acid accumulation and reduced fatty acid oxidation in HepG2 cells. This promotion of lipogenesis was associated with upregulation of SREBP1c and its downstream target genes, FAS. Inhibition of fatty acid oxidation was associated with downregulation of phosphorylation of AMPKα and ACC, as well as downregulation of CPT1 expression. Consistent with *in vitro* results, hepatic pathology *in vivo* showed that continuous administration of 5-PAHSA for 1 month in db/db mice caused liver steatosis, suggesting that 5-PAHSA treatment may induce liver damage in db/db mice. As a primary metabolic organ in human body, the liver plays a key role in the regulation of lipid and glucose metabolism. Dysregulation of hepatic lipid metabolic pathways contributes to the development of insulin resistance (Farese et al., 2012; Quiroga et al., 2012). Liver steatosis induced by 5-PAHSA treatment may one of the reasons that result in failure of glucose control in db/db mice.

Metabolic inflammation in tissue can interfere with insulin action through inhibiting insulin signaling pathway (Nie et al., 2017). We observed that 1 month of 5-PAHSA administration in db/db mice induced inflammatory responses, which may indicate a possible cause for the observed negative effects on insulin resistance. Specifically, serum IL-1α, CPR, and TNF-α were significantly higher in 5-PAHSA treated db/db mice than in db/db control mice. Hepatic pathology showed that continuous 5-PAHSA treatment in db/db mice aggravated hepatic inflammation. In addition, the levels of CPR and TNF-α in liver were significantly higher in 5-PAHSA treated db/db mice. These results were contrary to the report by Yore et al. (2014), which showed that oral administration of 5-PAHSA in HFD mice for 3 days improved adipose tissue inflammation by reducing the levels of macrophages that were positive for the proinflammatory cytokines TNF-α and IL-1β. Moreover, chronic 5-PAHSA treatment in chow-fed and HFD-fed mice reduces adipose inflammation (Syed et al., 2018). Based on this, we hypothesized that discrepancies between findings may be due to different animal models, since db/db mice had higher hyperglycemia. And our *in vitro* results supported the hypothesis.

In an inactive form, NF-κB is composed of a p65-p50 heterodimer bound with IκBα in the cytoplasm. After cellular stimulation, IκBα is degraded after phosphorylation and NF-κB is released to become the bioactive form. Active NF-κB is then translocated into nucleus and adjusts transcriptional activity of target genes, such as MCP1 and IL-6 (Baldwin, 1996). In the normal glucose condition, 5-PAHSA treatment inhibited inflammation via downregulation of IκBα phosphorylation and NF-κB phosphorylation, prevented IκBα degradation, NF-κB activation and then decreased MCP1 and IL-6 secretion. However, hyperglycemia impaired the anti-inflammatory effects of 5-PAHSA via promoting IκBα degradation and activating NF-κB pathway. Furthermore, since 5-PAHSA treatment caused a systemic inflammatory response in db/db mice, and chronic low-grade inflammation contributes to obesity-related insulin resistance (Odegaard and Chawla, 2013; Castoldi et al., 2015), our findings may provide insight into the exacerbated insulin resistance in db/db mice.

## Conclusion

We studied the effect of repeated 5-PAHSA treatment on glucose and lipid metabolism in db/db mice. Our results indicate that continuous 5-PAHSA treatment for 1 month might increase insulin resistance, promote lipid accumulation in the liver, and induce inflammatory responses. In mechanism, hyperglycemia impaired 5-PAHSA action by inhibiting the AMPKα signaling pathway and promoting NF-κB-mediated inflammation. In our future work, we plan to modify 5-PAHSA to further provide therapy for metabolic disease.

## Author Contributions

Y-MW and N-YF conceived the study. Y-MW and H-XL contributed to methodology. Y-MW investigated the study and wrote the manuscript. N-YF acquired funding, contributed to resources, and supervised the study.

## Conflict of Interest Statement

The authors declare that the research was conducted in the absence of any commercial or financial relationships that could be construed as a potential conflict of interest.
